# 3D Printed MEMS Technology—Recent Developments and Applications

**DOI:** 10.3390/mi11040434

**Published:** 2020-04-20

**Authors:** Tomasz Blachowicz, Andrea Ehrmann

**Affiliations:** 1 Institute of Physics-Center for Science and Education, Silesian University of Technology, 44-100 Gliwice, Poland; tomasz.blachowicz@polsl.pl; 2 Faculty of Engineering Sciences and Mathematics, Bielefeld University of Applied Sciences, 33619 Bielefeld, Germany

**Keywords:** 3D printing, microelectromechanical systems (MEMS), microelectronics, microfluidics, microsensors, microactuators

## Abstract

Microelectromechanical systems (MEMS) are of high interest for recent electronic applications. Their applications range from medicine to measurement technology, from microfluidics to the Internet of Things (IoT). In many cases, MEMS elements serve as sensors or actuators, e.g., in recent mobile phones, but also in future autonomously driving cars. Most MEMS elements are based on silicon, which is not deformed plastically under a load, as opposed to metals. While highly sophisticated solutions were already found for diverse MEMS sensors, actuators, and other elements, MEMS fabrication is less standardized than pure microelectronics, which sometimes blocks new ideas. One of the possibilities to overcome this problem may be the 3D printing approach. While most 3D printing technologies do not offer sufficient resolution for MEMS production, and many of the common 3D printing materials cannot be used for this application, there are still niches in which the 3D printing of MEMS enables producing new structures and thus creating elements for new applications, or the faster and less expensive production of common systems. Here, we give an overview of the most recent developments and applications in 3D printing of MEMS.

## 1. Introduction

Microelectromechanical systems (MEMS) are miniaturized devices combining electric and mechanical functions. Typical MEMS are, e.g., pressure and gyro sensors, accelerometers, or ink jet heads [1]. MEMS devices are often based on silicon (Si) [2,3]. Nevertheless, in the last decades, polymers were used in MEMS as well, e.g., polydimethylsiloxane (PDMS) for microfluidic devices [4], parylene for valves and sensors [5,6], or epoxy for micromanipulators [7].

Three-dimensional (3D) printing, on the other hand, works typically with polymers or metals and is recently not only applied for rapid prototyping, but also for the rapid production of individual parts or objects that could not be produced with other technologies [8,9]. Typical polymers used in the Fused Deposition Modeling (FDM) technology are acrylonitrile butadiene styrene (ABS), poly(lactic acid) (PLA), polyamide (“nylon”), or polycarbonate [10], while other technologies allow for printing different polymers. The disadvantages of most 3D printing technologies are relatively low mechanical properties, as compared to objects prepared from other technologies [11], which is why sometimes combinations of 3D printed parts with differently prepared objects are suggested [12,13].

**Figure 1:**
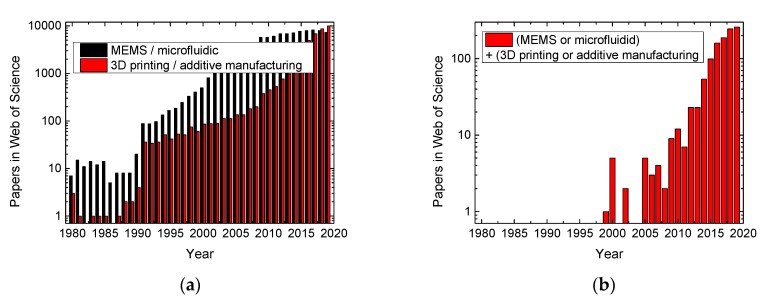
Number of papers found in the Web of Science, dealing with (**a**) microelectromechanical systems (MEMS) or microfluidic systems, and 3D printing or additive manufacturing, respectively; (**b**) the combination of MEMS or microfluidic and 3D printing or additive manufacturing. Data from the Web of Science, accessed on 31/03/2020.

As visible in Figure 1, both aforementioned technologies have emerged during the last decades (Figure 1a). MEMS have been reported in the scientific literature since 1980, while it took 10 more years until scientific research in 3D printing started and another decade until the first study on 3D printing of MEMS was reported. In spite of the additional degrees of freedom offered by 3D printing, on average, less than 1% of the studies dealing with 3D printing concentrate on MEMS. This is in contrast to the possible advantages of 3D printing in MEMS production, especially related to avoiding problems with an undesired underetching of 3D structures related to misalignments of the anisotropic etch pattern [14,15,16]. It can be expected that 3D printing methods allow for tailoring 3D shapes in the desired way, making the structures more reliable when the process parameters are properly adjusted.

On the other hand, 3D printing processes cause new challenges. The feature sizes that can be reliably reached depend on many parameters, such as the mechanical properties of the printer, the accuracy of stepper motors, but also on the technology used and the material applied for printing. Recently, freestanding polycrystalline copper pillars with diameters between 250 nm and 1.5 µm were reported as result of a 3D electrochemical deposition technique [17], as well as the direct laser writing of 3D submicron pillars [18], while common 3D printing techniques often are limited to the range of 100 µm resolution, combined with roughness or waviness in the order of several 10 µm, which necessitates chemical or heat-based post-treatment [19]. Table 1 gives an overview of typical resolutions reported for different 3D printing technologies. These typical minimum feature widths and printing speeds that can be reached depend on the chosen 3D printing technologies [20].

Another problem is the well-known thermal shrinkage occurring in polymer-based 3D printing processes [30,31] as well as after sintering green ceramics or during printing metal objects [32,33].

Here, we give an overview of recent research on 3D-printed MEMS, possible applications of such systems, as well as advantages and challenges connected with this combination of two modern technologies. Before, the most important 3D printing techniques are explained in brief.

## 2. Typical 3D Printing Techniques

Photolithography can be used by preparing a photomask, a glass plate, or plastic film coated with a non-UV-transparent film. The mask is placed on a photoresist on a silicon wafer, and this photoresist is not exposed to UV light through the open areas of the mask. Depending on the photoresist, either the exposed or the non-exposed areas form the pattern, while the residual photoresist is washed of the wafer, in this way building a master mold [34].

While this technique is well established to prepare three-dimensional objects, it is not necessarily meant when “3D printing” is mentioned. Instead, stereolithography is more often connected with the term “3D printing”. This technique is used to print light-sensitive materials from a polymer solution by using a laser to cross-link the polymer at desired positions. It can even be applied for the bioprinting of hydrogels [25].

An even more sophisticated way of using light to cross-link a UV-sensitive material is the two-photon or multi-photon polymerization [27,28]. These techniques need a tightly focused laser beam on a defined volume of the photosensitive polymer, enabling the material to absorb two or more photons simultaneously to reach an excited state. As usual in such second-order processes, its strength is proportional to the squared light intensity. This is why only in the very small focus volume of the laser can the process take place, opposite to the usual single-photon absorption used in stereolithography.

Selective laser sintering again uses a laser to build a 3D object layer by layer, but opposite to the aforementioned stereolithography not to photocure a resin, but rather to fuse small particles of thermoplastic powders, metals, or ceramics [21,22].

Inkjet printing belongs to the techniques that allow for printing biological material such as bioink or hydrogels [24]. Small ink droplets are dispersed on a substrate. Since typical inks have low viscosities; only relatively small 3D structures can be printed, typically in the order of magnitude of some 10 micrometers.

Fused deposition modeling (FDM) belongs to the well-known 3D printing methods since these printers are often available at low cost and are easy to use, but these basic versions are not very accurate. Generally, in this technique, a polymer is molten and pressed through a nozzle to be deposited along defined paths so that 3D structures are formed layer by layer [23].

Besides these often used technologies, there are several others, which are in most cases based on a layer-by-layer setup of a 3D structure. While typical minimum feature sizes are given in Table 1 for several 3D printing techniques, the maximum feature sizes depend on several parameters. Most techniques, besides photolithography, enable in principle printing objects of some centimeters to some 10 cm. The limiting factor is often the time, which depends not only on the technique but also on the desired printing quality or resolution, the chosen printing material, whether a newer or older printer is used, etc. On the other hand, most technologies need a printing bed or have similar size restrictions that cannot be overcome for a specific printer model. Thus, their values depend on too many parameters to be given generally for a certain technique.

## 3. 3D Printed Microfluidic Devices

The idea of 3D printing different parts of or full microfluidic devices was firstly mentioned in 2013. Leary et al. developed nanoparticles as contrast agents and investigated the possibility of moving them in MEMS structures used as “organs-on-a-chip”, including microchannels. In these MEMS channels, human cancer cells and normal cells were grown, and the detectability of small tumors by applying the superparamagnetic nanoparticles was investigated. Here, the authors suggested 3D printing of more complex 3D structures to overcome the limits of the 2D MEMS channels [35].

In 2014, Lifton et al. investigated possibilities to use 3D printing in MEMS technology and concluded that microfluidics and “labs-on-a-chip” would be the most suitable devices to be 3D printed due to the relatively large minimum feature size of 3D printing in the range of 50–500 µm. They compared stereolithography, micro-stereolithography, PolyJet, selective laser sintering, fused deposition modeling, and two less known technologies and suggested concentrating on enhancing the printing resolution toward a range of 1–10 µm to enable the utilization of 3D printing for a broader range of possible MEMS devices [36].

The next sub-sections will give some general remarks on the possible toxicity of the materials used for 3D printing and describe more recent developments in 3D-printed microfluidics, sorted by the respective printing technologies.

### 3.1. General Remarks on Toxicity of Materials for Biotechnological Applications

Concerns regarding the possible toxicity of typical 3D printing polymers used in the FDM technology, but also in stereolithography (SLA) and multi-jet modeling (MJM) were raised in 2015. The authors suggested for biotechnological applications, such as lab-on-a-chip, that polymers should be carefully selected to avoid erroneous results due to not taking into account a possibly reduced biocompatibility of the device [37].

This problem was also mentioned by other authors. Beckwith et al. underlined not only the high resolution, but also the noncytotoxicity of their 3D-printed microfluidic device prepared by SLA, which was used to investigate tumor fragments from biopsy samples [38]. SLA and material-jetting processes were used to prepare molds and afterwards positive replicas by soft lighography and poly(dimethylsiloxane) (PDMS) molding. Using zebrafish for the biotoxicity test, Fuad et al. found no toxicity in replicas from both 3D printing processes [39].

Conductive PLA, including graphite, was found to be biocompatible [40], while Zhu et al. had already shown the same for pure PLA [36]. On the other hand, for typical 3D printing materials, especially commercially available stereolithographic resins, toxic substances were found in diverse photoinitiators, photopolymers, and other compounds used in SLA resins as well as high growth inhibition or mortality in biocompatibility assessments of objects printed with different resins [41].

### 3.2. Photolithography

de Araujo et al. prepared microfluidic devices to create microbubbles, using 3D printing by an OBJET EDEN 250 printer which enables the printing of round channels with a diameter of 0.3 mm. The waxy support used along the channels had to be dissolved and mechanically removed from the channels after printing. In this way, it was possible to prepare microbubbles of very homogenous dimensions, with a standard deviation below 1% [34,42].

For a more often used microfluidic technology, the isothermal titration calorimetry (ITC), Jia et al. used 3D-printed microfluidic devices. Their microdevice combined the 3D-printed microfluidic structure with a polymer MEMS thermoelectric sensor to allow for ITC measurements of biomolecular interactions. Again, the 3D-printed device was found to work leak-free, in addition to the possibility of using non-permeable material and the geometric flexibility [43].

### 3.3. Two-Photon and Multi-Photon Polymerization

To prepare much smaller features, the so-called two-photon polymerization technique can be used, which enables preparing structures of dimensions below the diffraction limit, partly below 100 nm [44,45,46]. For this technique, usually ultrashort laser pulses are applied.

The polymer material used for this 3D printing process can be, e.g., chemically modified zirconium-based sol–gel composites [44], photoinitiators with highly toxic properties or modified ones with reduced or vanishing toxicity, such as riboflavin and triethanolamine [45], and a broad range of other materials for applications in optics, photonics, electronics, or biotechnology [46]. This technique was also suggested in different patents dealing with microfluidics [47].

### 3.4. Inkjet 3D Printing

An unusual application of microfluidics was reported by Walczak et al. [48]. They used the inkjet 3D printing of a microfluidic device with embedded force sensors to monitor the seed growth and axial growth forces of *Lepidum sativum* (garden cress) seeds, which are used in diverse plant growth investigations [49] and found growth forces around 50 mN for the root and 500 mN for the stalk [48].

### 3.5. Metal Additive Manufacturing

Huang et al. searched a solution for another problem. Since silicon-based microfluidic devices are often problematic at high temperatures where fluidic interconnects may be leaking, they used binder jet printing, which is a form of metal additive manufacturing. While such metal objects are usually highly porous and thus not suitable for application in microfluidics, the addition of boron nitride supported the sintering process and thus reduced the porosity, resulting in leak-free fitting even at high operating pressures [50].

### 3.6. Preparing Molds by 3D Printing Methods

Dinh et al. used 3D printing in combination with molding and plasma-assisted bonding to prepare a highly effective microfluidic active heating and cooling module, which was suggested for the large production of SiC power nanoelectronics [51].

A Venturi flowmeter was 3D printed by Adamski et al., combining a commercially available MEMS sensor with a 3D-printed microfluidic structure. Here, microchannel widths of 400–800 µm were reached [52].

Villegas et al. used PDMS molding, similar to Fuad et al. mentioned above [38], but they concentrated on reducing the surface roughness of the PDMS microfluidic channels from 2 to 0.2 µm by coating the mold with a fluorinated-silane and afterwards tethering an omniphobic lubricant to this adhesion layer [53].

### 3.7. Combining 3D Printing with Other Technologies

**Figure 2:**
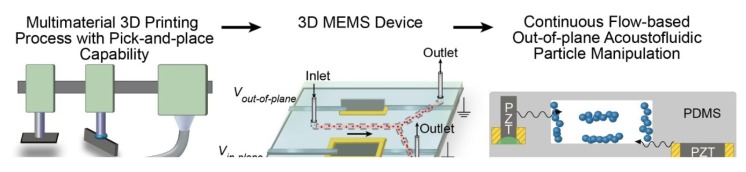
Combination of 3D printing and pick-and-place functionality to produce 3D MEMS devices used for acoustofluidic particle manipulation. Reproduced with permission from [54]. Copyright © The Royal Society of Chemistry 2018.

Cesewski et al. went one step further and combined a common 3D printing process with robotic handling in the form of an integrated pick-and-place functionality (Figure 2). In this way, they could assemble 3D printed forms with piezoelectric chips, which were used to produce multiple resonant modes to generate bulk acoustic waves, which could again be used to manipulate suspended particles [54].

Another combination of different technologies was suggested by Tamura and Suzuki, using 3D printing and photolithography to prepare the mold of a microfluidic device with micro- and millimeter structures. They showed that the process combination resulted in higher patterning accuracy than pure 3D printing [55].

The common MEMS fabrication technology of a boiler was combined with capillary channels with the 3D printing of a superheater. While the boiler alone could capture 2/3 of the incoming thermal energy, the addition of the superheater increased this value by 10%, in this way increasing thermal energy scavenging [56].

## 4. 3D-Printed Microelectromechanical Systems (MEMS) Sensors

The idea to use a 3D printing technology, in this case patterned 3D microstructures produced by an electrodepositable photo resist, in MEMS sensors was already mentioned in the scientific literature in 2007 [57]. For the special case of freeform helical microstructures, Farahani et al. investigated in 2014 which 3D printing methods were most suitable and found a high performance of UV-assisted and solvent-cast 3D printing methods, however, with a limited range of usable materials [58].

However, afterwards, it took some time until the first 3D printed MEMS sensors were reported in the literature. Some important chemical and physical sensors are described here.

### 4.1. Chemical Sensors

3D printing was used to produce a resonant gas cell, which was combined with commercially available fiber optics and a MEMS microphone sensor, in this way creating a miniaturized photoacoustic trace gas sensor [59]. Here, the sensor itself was not 3D printed, but combined with a 3D printed gas cell.

A breath analyzer, measuring CO_2_ and oxygen in the exhaled breath of humans, was partly 3D printed. Equipped with a thermopile detector and a MEMS infrared emitter, even low CO_2_ concentrations could be detected, and results similar to commercially available breath-by-breath sensors were achieved [60].

Ilke et al., on the other hand, integrated MEMS or electret microphones into 3D printed photo-acoustic gas sensors for the mid-infrared. These sensors could be used to detect methane with detection limits of 90 ppm for the electret microphone and 182 ppm for the MEMS microphone, applying similar integration times around 260 s [61].

### 4.2. Physical Sensors

Similarly, Valyrakis et al. used 3D printing to prepare a waterproof hollow spherical particle in which a three-axial gyroscope and accelerometer were embedded to measure hydrodynamic forces on a coarse particle during entraining from the riverbed, aiming at understanding sediment transport [62]. Shen et al. also used 3D printing to prepare a fixture for a piezoresistive MEMS flow rate sensor. Both were bonded and integrated on an intravenous tube, allowing for measuring the flow rate in this tube [63]. Similarly, Raoufi et al. developed a MEMS flow sensor embedded in a 3D printed semicircular channel, aiming to enhance current artificial vestibular systems [64].

A 3D-printed housing for an airborne nanoparticle concentration sensor was prepared using a MEMS-based particle growth chip that grows nanoparticles to micro-sized droplets by condensation, in combination with a miniaturized optical particle counter based on a light-scattering method. This system was able to detect nanoparticles of 12.4 nm diameter and showed only small deviations from a reference instrument in high and low concentration environments [65].

A miniaturized transmission electron microscope (TEM) was suggested by Krysztof et al. They prepared a 3D-printed polymer holder for mounting the field emission cathode and electron optics columns and showed effective emission and focusing of the electron beam inside a vacuum chamber as the first step toward a MEMS-TEM [66].

Das et al. used 3D printing in the form of electrohydrodynamic ink jetting to produce PEDOT:PSS strain gauge sensors on kapton and silicone substrates. Comparing COMSOL simulations with the experimental results showed sufficient agreement. The authors suggested using such a system as future piezo-resistive robot skin [67]. However, in a newer work, a novel wet lift-off photolithographic technique was proposed for patterning the poly(3,4-ethylenedioxythiophene) polystyrene sulfonate (PEDOT:PSS) base layer due to the partly problematic electrohydrodynamic jetting technology [68]. Direct ink writing was suggested by Yang et al. instead to pattern 3D conductive circuits on flexible substrates [69].

3D printing was also used to prepare spiral-shaped acoustic resonators, as inspired by the human ear, serving as frequency-selective MEMS microphones. Using these MEMS sensors, they could strongly increase the frequency sensitivity of the investigated resonance frequency of 430 Hz [70].

Tiller et al. used the 3D printing process of digital light processing to prepare a piezoelectric acoustic sensor from piezoelectric and conductive parts with mechanically sensitive membranes of thicknesses as low as 35 µm, allowing for tunable resonant frequencies [71].

Capacitive MEMS vibration sensors were produced by a high-current plasma focused ion beam (FIB) technique and compared with sensors prepared by the common lithography process. While the resonance frequency differed by only 4%, the fabrication time could be reduced by approximately 80% [72].

**Figure 3:**
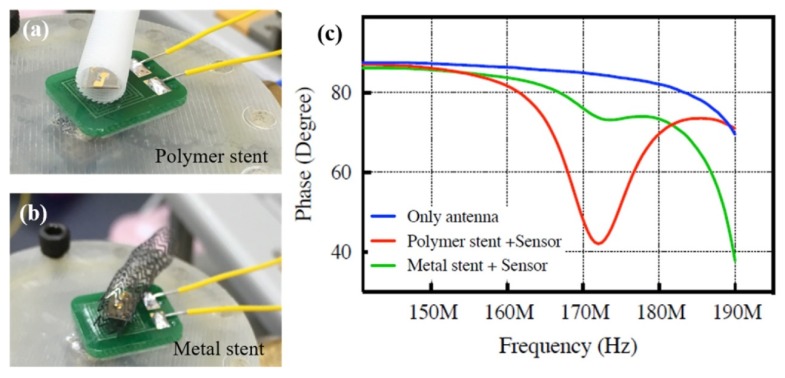
Wireless pressure sensors integrated in (**a**) polymer and (**b**) metal stents; (**c**) sensitivity analysis of these combinations. Reproduced with permission from [73]. Copyright © Elsevier 2019.

Park et al. integrated a MEMS pressure sensor based on an inductor–capacitor resonant circuit into metallic and polymer 3D-printed stents for blood vessels. As shown in Figure 3, the sensitivity of the pressure sensor in the polymer stent was much higher than that of the metal stent system, allowing for real-time monitoring [73].

Recently, Wang et al. developed a 3D-printed MEMS device, using projection micro-stereolithography, which could be used for in situ tensile tests of micro- or nanowires. This device was used to determine the tensile behavior of SiC nanowires inside a scanning electron microscope (SEM) as well as lead zirconate titanate microwires under an optical microscope, in this way demonstrating micro- and nanomechanical characterization [74].

## 5. 3D-Printed MEMS Actuators

Identical to sensors, 3D-printed MEMS actuators were firstly suggested in 2007 [57]. Again, the first scientific reports on experimental investigations of such actuators were published several years later. Here, some of the proposed 3D-printed MEMS actuators are depicted, which were sorted according to their applications.

### 5.1. Switches

**Figure 4:**
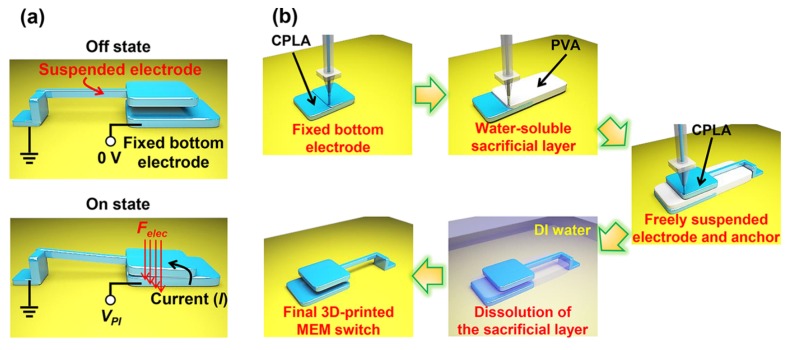
(**a**) Concept of 3D-printed MEMS switch, showing the pull-in phenomenon due to electrostatic forces on the suspended electrode when a voltage is applied to the fixed bottom electrode; (**b**) 3D printing process. Reproduced with permission from [75]. Copyright (2018) American Chemical Society.

A 3D-printed MEMS switch was developed by Lee et al. Using the FDM printing of conductive PLA and poly(vinyl alcohol) (PVA) as water-soluble support, a switch with good electromechanical properties, abrupt switching, and a very high on/off current ratio above 10^6^ was realized. The function and printing process are depicted in Figure 4 [75].

### 5.2. Vibration Actuator

Xie et al. used 3D printing for vibrational tactile actuators for blind people. They combined piezoelectric extensional actuators vibrating in-plane with a scissor mechanism, forming a triangle between two in-plane points and one point above the plane. The oscillations of the two in-plane points in opposite directions led to oscillations of the third point rectangular to the plane. Placing the third point not too high above the plane resulted in conversion of small in-plane actuations into higher-amplitude out-of-plane vibrations, which can be sensed by a finger. These scissor amplifiers were produced by conventional photoresist and 3D printing. However, tests with volunteers showed that the minimum and the comfortable forces needed for the detection of the vibrations was slightly higher for the 3D-printed scissors, which was attributed to sensing being dominated by vibrational amplitudes rather than by forces [76].

### 5.3. Aeronautics and Astronautics

**Figure 5:**
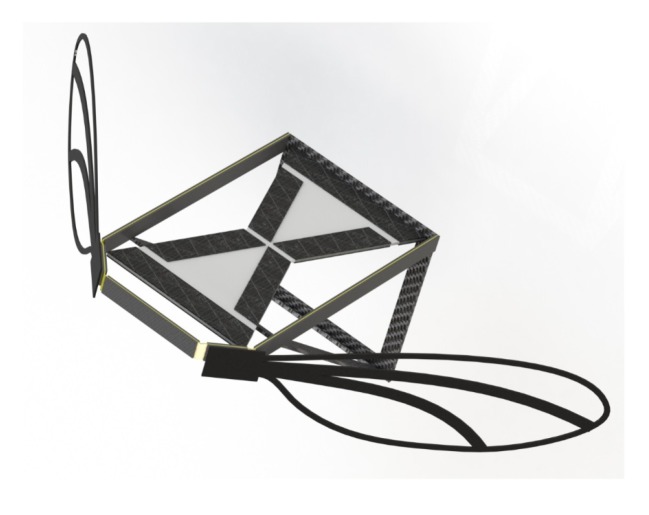
Concept design of a micro aerial vehicle, composed of MEMS and 3D-printed parts. Reproduced with permission from [77].

Combining MEMS dielectric elastomer actuators with a 3D-printed wing skeleton on which a fine Mylar film was glued was suggested to create micro aerial vehicles. While the wings were optimized using computational fluid dynamic simulations, the dielectric elastomer actuator was used due to its high work density, the ability to work at high frequencies, and the easy, low-cost production. Both parts were connected with a four-bar mechanism with a pivot point at the wing base. The design concept is depicted in Figure 5 [77], showing one of the possibilities of combining MEMS with 3D printing to produce miniaturized aerial vehicles.

Khandekar et al. used 3D printing to prepare microthrusters for microsatellites, which can be used for very low thrust in a defined direction for course corrections of nano and microsatellites. Printing ceramic polymer composites, micro-thrusters with sufficient nanomechanical properties to propel nano and microsatellites were developed [78].

### 5.4. Nanopositioning

Fiaz et al. developed cantilevers for MEMS using the electron beam melting of a Ti alloy. The material was chosen due to the good biocompatibility of Ti, its strength, and its corrosion resistance. Comparing these cantilevers with bulk metal ones, the printed cantilevers were softer with a slightly smaller Young’s modulus than the bulk material, but they also reached large maximum displacements of nearly 50 µm at resonance frequencies around 1850 Hz and thus relatively fast and accurate positioning of a stage [79].

Using an SLA printer with different commercially available and self-formulated resins in combination with the electrodeposition of different metals, Bernasconi et al. prepared another magnetic actuator. By coating the 3D-printed cantilever with magnetic metals, the cantilever could be deflected by an external magnetic field. The pseudo-linear correlation between the field and deflection was measured and modeled [80].

Magnetic NdFeB microparticles were embedded instead in a nylon 12 matrix to prepare a magnetic actuator, which reached a maximum displacement of 50 µm [81].

Similarly, electrothermal actuators were produced by Fogel et al. They used the 3D printing technology of laser-induced forward transfer (LIFT) to prepare fully metallic microdevices from structural and sacrificial metals, allowing preparing a free-standing structure on the support material. This actuator was deflected by a current, which was applied for a certain pulse duration with a maximum of 1 s [82].

Ertugrul et al. compared the aforementioned two-photon polymerization with projection micro-stereolithography in the case of an electrothermal microactuator. They found that the two-photon polymerization was advantageous since it could produce smaller and more complex, non-symmetric structures [83].

### 5.5. Macro-Positioning

3D printing was used to prepare a hybrid finger composed of hard and soft polymer, equipped with micropumps to enable hydraulic motion control. These MEMS micropumps worked with an electro-conjugate fluid and were found to have sufficient output density to drive the hybrid finger [84].

## 6. Conclusions

After the first ideas of 3D printing MEMS approximately 20 years ago, much progress was achieved in combining these technologies. Especially microfluidic systems, but also some MEMS sensors and actuators can nowadays be realized by diverse 3D printing technologies. New additive manufacturing techniques, such as the two-photon polymerization technique, allow for preparing smallest features with dimensions below 1 µm. For more established 3D printing techniques, new ideas emerged how to reduce the minimum feature size, in this way making 3D printing more and more suitable for MEMS fabrication.

Table 2 gives a short overview of 3D printing methods and their possible applications in MEMS, indicating the broad bandwidth of technologies and functions that can be reached by them.

Nevertheless, it seems that the research published on the combined technologies has passed its peak. Thus, we hope that this review will stimulate more researchers to investigate new possible applications, enabled especially by newly developed and future 3D printing techniques.

## Figures and Tables

**Table 1:** Resolutions of different 3D printing technologies, reported in the literature, sorted from larger to smaller minimum feature sizes.

Technology	Min. Feature Size	Material	Ref.
Selective laser sintering	<400 µm	Div. Polymers	[21,22]
Fused deposition modeling	200 µm	Diverse polymers	[23]
Robot dispensing	200 µm	Hydrogels	[24]
Stereolithography	30–70 µm	Photosensitive polymers	[25]
3D inkjet printing	28 µm	Photoresist	[24]
Resonant direct laser writing	1–4 µm	IP-Dip photoresist	[26]
Multiphoton absorption polymerization	1 µm	SU8 photoresist	[27]
Two-photon polymerization	0.28–1.5 µm	Photoresists	[28]
Direct laser writing	0.085–1.5 µm	Photoresists	[29]

**Table 2:** Possible applications of typical 3D printing technologies used for MEMS.

Technology	Possible Applications
Fused deposition modeling	Dielectric-conductive systems, switches
Micro-stereolithography	In situ tensile tests of micro- or nanowires, electrothermal microactuator
Stereolithography	Microfluidic devices, conductive parts, molds, cantilevers, magnetic actuators
3D inkjet printing	Microfluidic devices, Venturi microflowmeter, conductive structures, strain gauge sensors
Multiphoton absorption polymerization	Microfluidic devices, photonic crystals, nanophotonic devices
Two-photon polymerization	Microfluidic devices, electrothermal microactuator
Binder jet printing	Microfluidic devices, in-line injection of volatile organic compounds
